# Clinical evaluation of modified invaginated pancreaticojejunostomy for pancreaticoduodenectomy

**DOI:** 10.1186/s12957-020-01851-6

**Published:** 2020-04-15

**Authors:** Dong Wang, Xiao Liu, Hongwei Wu, Kun Liu, Xiaona Zhou, Jun Liu, Wei Guo, Zhongtao Zhang

**Affiliations:** Department of General Surgery, Beijing Friendship Hospital, Capital Medical University; Beijing Key Laboratory of Cancer Invasion and Metastasis Research & National Clinical Research Center for Digestive Diseases, 95 Yong-an Road, Xi-Cheng District, Beijing, 100050 China

**Keywords:** Modified invaginated pancreaticojejunostomy, Mucosa-to-mucosa anastomosis, Pancreaticoduodenectomy

## Abstract

**Background:**

Pancreaticoduodenectomy (PD) remains the major curative operation for malignant neoplasm of pancreas or cancerous tumors near the pancreas. Despite advancements in recent years, the postoperative recurrence rate of these neoplasms and tumors remains high. Moreover, overall morbidity remains high due to clinically relevant postoperative pancreatic fistula (POPF).

**Methods:**

To compare the clinical outcomes of modified invaginated anastomosis and mucosa-to-mucosa anastomosis, this retrospective study included 343 patients who underwent PD from January 2008 to January 2019 at Beijing Friendship Hospital, Capital Medical University. The patients’ general conditions and disease status were preoperatively evaluated. The surgical procedure was recorded, and operative management was appropriately performed.

**Results:**

Compared with mucosa-to-mucosa anastomosis, modified invaginated anastomosis resulted in a higher intraoperative blood transfusion rate (*P* < 0.001) and lower hospitalization expenses (*P* = 0.049). However, no significant differences were found in operation time (*P* = 0.790), intraoperative bleeding (*P* = 0.428), postoperative recovery exhaust time (*P* = 0.442), time to normal flow of food (*P* = 0.163), and hospitalization time (*P* = 0.567). Operation time was a risk factor for POPF (odds ratio 1.010; 95% confidence interval 1.003–1.016; *P* = 0.003). The incidence of pancreatic fistula (grades B and C) was lower in the patients who underwent modified invaginated anastomosis (14.1%) than in those who underwent mucosa-to-mucosa anastomosis (15.3%). The operation time was greater in the POPF group than in the non POPF group among the patients who received modified invaginated anastomosis (*P* = 0.003) and mucosa-to-mucosa anastomosis (*P* = 0.002).

**Conclusion:**

Modified invaginated pancreaticojejunostomy for PD resulted in a decreased incidence of POPF; it may serve as a new approach for PD while managing patients who have undergone PD.

## Introduction

Surgical resection for periampullary diseases is an important treatment modality and primarily includes total pancreatectomy (TP) and pancreaticoduodenectomy (PD) [1]. Unlike TP, which results in the permanent insufficiency of pancreatic endocrine and exocrine function, PD is more feasible and still remains a major curative operation for malignant neoplasm of pancreas and cancerous tumors near the pancreas [2–5]. PD holds a fairly high surgical risk, thus presenting challenges to the surgeon [6–8]. Despite advancements in surgical procedures, interventional radiology, perioperative management, and anesthesia techniques in recent years, the postoperative recurrence rate of these neoplasms and tumors remains high at up to 40%, which is higher than that of other gastroenterological cancers [3, 9, 10]. Moreover, overall morbidity is still high due to clinically relevant postoperative pancreatic fistula (POPF) [11, 12].

Several modified approaches for PD have been proposed and studied. Kawabata et al. reported a modified total mesopancreatoduodenum excision for PD [13], which greatly improved the overall survival (65.3% at 1 year and 35.2% at 3 years, respectively). Some studies reported a modified Blumgart pancreaticojejunostomy for PD and compared it with other techniques [6, 14], showing its great advantage with a low rate of morbidity and mortality but with simplified procedures that can be easily put into practice. Moreover, Aghalarov et al. proposed a modified single-loop reconstruction after PD that could reduce the severity of POPF in high-risk patients [15], and thus leading to less major morbidity and mortality. Based on the experience at our center, a clinical evaluation of the modified invaginated pancreaticojejunostomy for PD was performed.

**Fig. 1:**
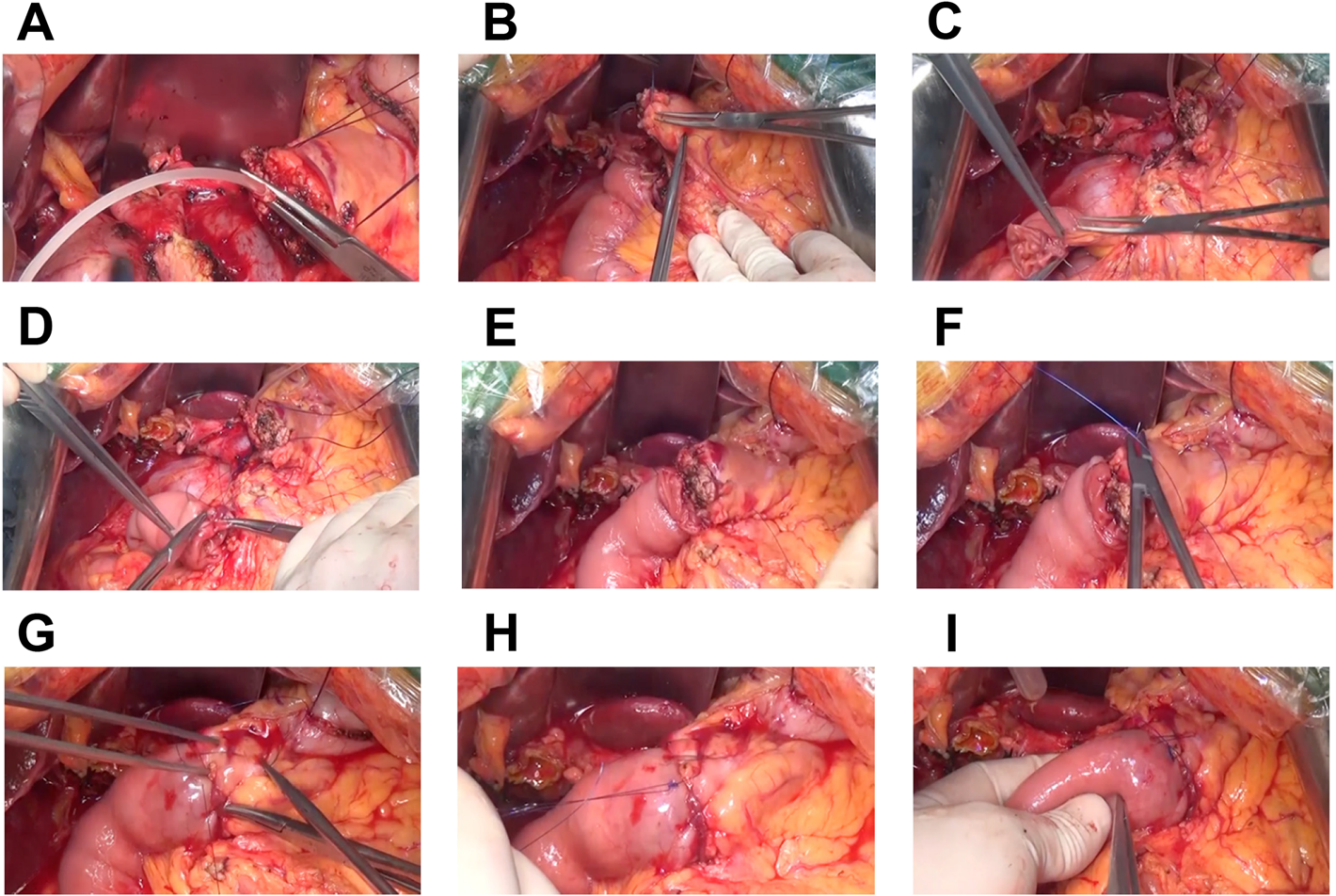
Schematic illustration of the modified invaginated pancreaticojejunostomy approach for pancreaticoduodenectomy. **a** Search the main pancreatic duct and insert the support catheter. **b** The posterior wall of the pancreaticojejunostomy was sutured from the lower edge of the pancreas. **c** The jejunum started from the mesentery margin and was sutured by the inversion of the plasma muscular layer. **d** The suture of the posterior wall of the anastomosis was performed under direct vision without tightening the sutures. **e** After the jejunum-side mucosa was embedded, the sutures were tightened together after the posterior wall anastomosis was completed. **f** Pancreas suture started from the pancreas. **g** The jejunum seromuscular layer suture and each needle must be tightened. **h** Knot to the lower edge of the pancreas and the end of the line. **i** Sleeve around the pancreas about 3 cm

In this study, the modified invaginated pancreaticojejunostomy method for PD used at our center has been introduced (Fig. 1) based on the clinical data collected in the past 10 years, and this modified method has been compared with mucosa-to-mucosa anastomosis for PD.

## Materials and methods

### Study participants

A total of 343 patients who underwent PD (R0 or R1 resection) from January 2008 to January 2019 at Beijing Friendship Hospital affiliated to Capital Medical University were included in this retrospective study. The exclusion criteria were as follows: patients aged less than 18 years, patients with abdominal and other systemic infections prior to surgery, patients receiving neoadjuvant therapy prior to surgery, patients with a history of abdominal surgery, uncooperative patients, and patients with incomplete clinical data.

### Preoperative treatment

Prior to surgery, the patients’ general conditions and disease status were evaluated, which included routine blood test, blood type examination, heart function examination, liver function tests, kidney function tests, coagulation function examination, infection screening, tumor marker examination, contrast-enhanced chest computed tomography (CT), plain abdominal pelvic contrast-enhanced CT combined with abdominal vascular three-dimensional reconstruction, and upper abdominal contrast-enhanced magnetic resonance imaging. Moreover, ultrasound endoscopy and biopsy were conducted. Percutaneous transhepatic cholangial drainage was performed in patients with total bilirubin levels exceeding 200 μmol/L, whereas liver protection treatment and an intramuscular injection of vitamin K were administered in patients with increased preoperative bilirubin levels and abnormal liver function. When specialized organ dysfunction combined with cardiovascular or cerebrovascular diseases was observed, specialists were consulted, and the treatment was adjusted accordingly.

### Surgical methods of modified invaginated pancreaticojejunostomy

The patients were placed in the supine position and were anesthetized by tracheal intubation, and a longitudinal incision was made in the upper abdomen for exploratory laparotomy. Patients without surgical contraindications underwent PD and lymph node dissection. When no active bleeding was observed in the surgical field, the surgery was initiated (Fig. 1). To locate the main pancreatic duct, the pancreatic stump was dissociated by 2–3 cm, and a stent tube that matched its diameter (at least 3 cm) was inserted at a depth of approximately 10 cm (Fig. 1a). The pancreatic stump was continuously stitched from the lower margin to the seromuscular layer of the posterior jejunal stump with a 3-0 prolene suture (Fig. 1b). Then, the suture was tightened with 3–4 stiches on the posterior wall of the anastomosis, and each stitch was tightened at the anterior wall from the upper margin (Fig. 1c). The needle was inserted from the tail of the pancreas at approximately 2 cm from the broken end. The pancreaticojejunostomy was competed using the invagination approach, i.e., the 2–3-cm pancreatic stump was invaginated into the inverted jejunal stump (Fig. 1d). One week after suturing, the prolene suture was moderately tightened to ensure that the end of the pancreas was located in the jejunum at a depth of 2–3 cm. Biliary and gastrointestinal anastomosis was performed after completion of the pancreaticojejunostomy. Finally, the conventional indwelling silicone drainage tube was placed on the omental capsule.

### Postoperative management

After fasting and rehydration, intravenous nutrition was administered to the patients.

The inflow volume, bowel sounds, routine blood tests, electrolyte examination, amylase concentration, and the amount and properties of the drainage fluid were monitored at least every other day for 7 days postoperatively. The patients were treated with antibiotics, proton pump inhibitors, and somatostatin. They were encouraged to walk when their vital signs were stable. The stomach tube was removed when the gastrointestinal decompression was less than 200 mL, with anus venting and bowel sound recovery. Normal flow of food was restored when the patient could drink a small amount of water.

### Definitions and diagnosis

The texture of the pancreas was determined by the surgeon through observation and palpation of the pancreatic tissue during the surgery and was classified as soft, tough, or hard. The diameter of the main pancreatic duct was measured by a pathologist based on the diameter of the pancreatic duct of the specimen. Data on surgery-related indicators, including operative time, intraoperative blood loss, and intraoperative blood transfusion, were obtained from the surgical records.

Data on postoperative complications, exhaust time, time to normal flow of food (porridge), hospitalization time, total hospitalization expenses, etc., were collected from the disease records and home management system.

Pancreatic fistula was diagnosed when the amylase concentration in the drainage fluid was more than 3-fold of the upper limit of the normal serum amylase concentration after ≥ 3 postoperative days and when it displayed corresponding clinical manifestations. Pancreatic grading was determined according to the definition of the International Study Group of Pancreatic Surgery (ISGPS). POPFs of grade B or C were considered significant.

Bile leakage was confirmed by either clinical observation of the drainage fluid or a drainage fluid bilirubin assay combined with clinical manifestations. Postoperative bleeding, including bloody fluid and blood in the abdominal drainage tube or gastrointestinal tube, was diagnosed when hemoglobin levels were significantly reduced on routine blood tests and when related changes in vital signs such as heart rate and blood pressure were observed.

Postoperative gastrointestinal dysfunction was defined as removal of the gastric tube at more than 7 days postoperatively, reintubation due to vomiting, or abdominal distension for 10 days postoperatively.

Abdominal infection was defined as postoperative chills, high fever, and abdominal distension for more than 24 h; it was diagnosed by positive results of drainage fluid culture, significantly increased white blood cell count, and intraabdominal fluid accumulation, as observed on ultrasound or CT.

All postoperative complications, except pancreatic fistula, were graded according to the Clavien–Dindo classification of surgical complications.

### Statistical analysis

Statistical data were analyzed using the SPSS 22.0 software (IBM, New York, USA). Quantitative data are expressed as mean ± standard deviation. Independent *t*-test was used to compare continuous variables between two groups as described in previous literatures [16, 17]. *P* values of < 0.05 were considered statistically significant.

## Results

Overall, 199 patients underwent modified invaginated pancreatoenterostomy, among which 114 were men and 85 were women. The average age of these patients was 57.2 ± 10.7 years (range, 19.0–85.0 years). Overall, 88 patients were diagnosed with a tumor or mass in the ampulla, and 111 patients were diagnosed with icterus.

Moreover, 144 patients underwent jejunum–pancreatic duct mucosa-to-mucosa anastomosis, among which 86 were men and 58 were women. The average age of these patients was 61.8 ± 8.9 years (range, 30.0–82.0 years). Overall, 82 patients were diagnosed with a tumor or mass in the ampulla, and 62 patients were diagnosed with icterus.

As shown in Table 1, the operation time, intraoperative bleeding, intraoperative blood transfusion rate, postoperative recovery exhaust time, time to normal flow of food, hospitalization time, and hospitalization expenses were compared between the two groups. The intraoperative blood transfusion rate was higher in the modified invaginated anastomosis group (1.1 ± 2.0) than in the mucosa-to-mucosa anastomosis group (0.7 ± 1.7) (*P* < 0.001). Similarly, the hospitalization expenses were higher in the modified invaginated anastomosis group (99867.4 ± 44456.0 RMB) than in the mucosa-to-mucosa anastomosis group (89728.9 ± 50519.67 RMB) (*P* = 0.049). However, no significant differences were found in the operation time (*P* = 0.790), intraoperative bleeding (*P* = 0.428), postoperative recovery exhaust time (*P* = 0.442), time to normal flow of food (*P* = 0.163), and hospitalization time (*P* = 0.567).

**Table 1:** Characteristics of pancreaticojejunostomy

	Modified invaginated anastomosis	Mucosa-to-mucosa anastomosis	*P* value
Number	199	144	
Sex			
Male (number)	114	86	
Female (number)	85	58	
Operation time (min)	187.0 ± 58.1	190.2 ± 49.0	0.790
Intraoperative bleeding (mL)	493.3 ± 449.2	519.0 ± 440.4	0.428
Intraoperative blood transfusion (U)	1.1 ± 2.0	0.7 ± 1.7	**< 0.001**
Postoperative recovery exhaust time (day)	4.0 ± 1.1	3.9 ± 1.5	0.442
Time to normal flow of food (day)	9.8 ± 3.4	10.3 ± 2.9	0.163
Hospitalization time (day)	29.9 ± 13.5	29.0 ± 13.7	0.567
Hospitalization expenses (RMB)	99867.4 ± 44456.0	89728.9 ± 50519.67	0.049

By analyzing the risk factors for pancreatic fistula (Table 2), operation time was identified as a significant risk factor (odds ratio (OR) 1.010; *P* = 0.003), whereas the other factors showed no statistical significance. In terms of pancreatic fistula (Table 3), 128 (64.3%) of the 199 patients who underwent modified invaginated anastomosis had grade A pancreatic fistula (biochemical fistula), 28 (14.1%) had grade B pancreatic fistula, and none of them (0%) had grade C pancreatic fistula. Furthermore, 89 (61.8%) of the 144 patients who underwent mucosa-to-mucosa anastomosis had grade A pancreatic fistula (biochemical fistula), 21 (14.6%) had grade B pancreatic fistula, and 1 (0.7%) had grade C pancreatic fistula. The percentage of meaningful pancreatic fistula (grades B and C) was lower in the modified invaginated anastomosis group (14.1%) than in the mucosa-to-mucosa anastomosis group (15.3%). Moreover, the highest amylase concentration on postoperative day 3 in patients with grades A (5493.3 ± 966.0 U/mL) and B (6817.9 ± 1423.7 U/mL) fistulae who received mucosa-to-mucosa anastomosis was higher than that in patients with grades A (3930.7 ± 639.3 U/mL) and B (5666.1 ± 1330.0 U/mL) fistulae who received modified invaginated anastomosis (*P* < 0.5).

**Table 2:** Risk factors for pancreatic fistula

	Odds ratio	95% Confidence interval		*P* value
		Lower	Upper	
Sex	1.177	0.706	1.961	0.533
Operation time	**1.010**	1.003	1.016	**0.003**
Hospitalization time	1.031	1.006	1.057	0.016
Time to normal flow of food	1.034	0.955	1.119	0.412
Intraoperative bleeding	1.000	0.999	1.000	0.322
Postoperative recovery exhaust time	0.857	0.734	1.000	0.050

**Table 3:** Incidence of pancreatic fistula and the highest amylase concentration on postoperative day 3

		Modified invaginated anastomosis	Mucosa-to-mucosa anastomosis	*P* value
Cases [number (%)]	None	43 (21.6%)	33 (22.9%)	0.003
	Grade A	128 (64.3%)	89 (61.8%)	
	Grade B	28 (14.1%)	21 (14.6%)	
	Grade C	0	1 (0.7%)	
Amylase concentration (U/mL)	None	126.6 ± 91.7	106.5 ± 83.4	0.328
	Grade A	3930.7 ± 639.3	5493.3 ± 966.0	0.023
	Grade B	5666.1 ± 1330.0	6817.9 ± 1423.7	0.037
	Grade C	-	3106	-

The pathology of the resected neoplasm was postoperatively evaluated (Table 4). In the modified invaginated anastomosis group, 71 had duodenal tumors, 56 had pancreatic tumors, 66 had lower bile duct tumors, and 6 had inflammatory lesions. In the mucosa-to-mucosa anastomosis group, 38 had duodenal tumors, 47 had pancreatic tumors, 49 had lower bile duct tumors, and 10 had inflammatory lesions. The incidence of duodenal tumors (*P* = 0.002) was higher and that of pancreatic tumors (*P* = 0.007) was lower in patients who underwent modified invaginated anastomosis than in those who underwent mucosa-to-mucosa anastomosis.

**Table 4:** Postoperative pathology of the modified invaginated anastomosis and mucosa-to-mucosa anastomosis groups

Pathological type	Modified invaginated anastomosis	Mucosa-to-mucosa anastomosis	*P* value
Duodenal tumor [number (%)]	71 (35.7%)	38 (26.4%)	0.002
Duodenal papillary adenocarcinoma (number)	38	21	
Duodenal ampullary adenocarcinoma (number)	15	9	
Duodenal adenoma (number)	14	6	
Duodenal neuroendocrine tumor (number)	4	2	
Pancreatic tumor [number (%)]	56 (28.1%)	47 (32.6%)	0.007
Pancreatic ductal adenocarcinoma (number)	32	26	
Pancreatic serous cystadenoma (number)	9	7	
Intraductal papillary mucinous neoplasm (number)	5	6	
Solid pseudopapillary tumor (number)	5	4	
Pancreatic neuroendocrine tumor (number)	4	4	
Pancreatic intraepithelial neoplasia (number)	1	0	
Lower bile duct tumor, [number (%)]	66 (33.2%)	49 (34.0%)	0.113
Bile duct adenocarcinoma (number)	62	44	
Bile duct adenoma (number)	4	5	
Inflammatory lesion (number)	6 (3.0%)	10 (7.0%)	0.317
Chronic pancreatitis (number)	5	8	
Bile duct inflammation (number)	1	2	

Furthermore, each group was divided into the non POPF (patients without a POPF) and POPF (patients with a POPF) subgroups, and the patients who underwent modified invaginated anastomosis in these subgroups were compared (Table 5). The operation time in the POPF group (224 min) was higher than that in the non POPF group (178 min) (*P* = 0.003). No significant differences were observed in age (*P* = 0.748), intraoperative bleeding (*P* = 0.684), preoperative albumin level (*P* = 0.727), preoperative hemoglobin level (*P* = 0.237), preoperative total bilirubin level (*P* = 0.299), incidence of soft pancreas (*P* = 0.881), and main pancreatic duct stricture (*P* = 0.793).

**Table 5:** Comparisons between the non POPF and POPF subtypes following the modified invaginated anastomosis approach

	Non POPF (*n* = 171)	POPF (*n* = 28)	*P* value
Age, years	57	55.8	0.748
Intraoperative bleeding (mL)	457	596	0.684
Operation time (min)	178	224	**0.003**
Preoperative albumin level (g/L)	36.4	36.6	0.727
Preoperative hemoglobin level (g/L)	126.3	129.2	0.237
Preoperative total bilirubin level (μmol/L)	95.7	82.6	0.299
Soft pancreas [number (%)]	130 [76%]	23 [82%]	0.881
Main pancreatic duct stricture [number (%)]	116 [68%]	20 [71%]	0.793

When the patients who underwent mucosa-to-mucosa anastomosis in these subgroups were compared (Table 6), the operation time was found to be greater in the POPF group (236 min) than in the non POPF group (185 min) (*P* = 0.002). The main pancreatic duct stricture was more severe in the POPF group (77%) than in the non POPF group (57%) (*P* = 0.024). No significant differences were observed in age (*P* = 0.782), intraoperative bleeding (*P* = 0.309), preoperative albumin level (*P* = 0.728), preoperative hemoglobin level (*P* = 0.197), preoperative total bilirubin level (*P* = 0702), or incidence of soft pancreas (*P* = 0.130).

**Table 6:** Comparisons between the non POPF and POPF subtypes following the mucosa-to-mucosa anastomosis approach

	Non POPF (*n* = 122)	POPF (*n* = 22)	*P* value
Age (year)	56.2	54.3	0.782
Intraoperative bleeding (mL)	467	591	0.309
Operation time (min)	185	236	**0.002**
Preoperative albumin level (g/L)	35.4	36.7	0.728
Preoperative hemoglobin level (g/L)	129.2	130.1	0.197
Preoperative total bilirubin level (μmol/L)	92.3	95.6	0.702
Soft pancreas [number (%)]	83 (68%)	19 (86%)	0.130
Main pancreatic duct stricture [number (%)]	70 (57%)	17 (77%)	**0.024**

## Discussion

The findings of the present study revealed that the technical point of this modified invaginated anastomosis is to initially use continuous suture and then finally tighten the suture with a certain reserved tension, which ensures even stress in the pancreatic tissue. Compared with the traditional technique, tightening each needle in the suture of the posterior wall of the anastomosis was not required until the posterior wall is sutured in the modified invaginated pancreaticojejunostomy, which means the approach could be achieved under direct vision, making the anastomosis more precise. Moreover, in present technique, the intestinal wall serous muscle layer of both the anterior and posterior walls were sutured to ensure the intestinal wall serous layer and the pancreatic serous layer were aligned, which is helpful for anastomotic healing. The stump of the pancreas was inserted into the intestinal canal 2–3 cm to ensure the tightness of the anastomosis, and this method is applicable to pancreas of various textures. In general, by unifying the method of suturing the anterior and posterior walls, present technique saves the operation time and is simpler and easier to operate for beginners.

Since the development of PD for more than 100 years, surgeons have improved the reconstruction methods and perioperative management strategies. Despite the dozens of digestive tract reconstruction approaches that have been proposed, the best surgical method for PD remains controversial to date. Although improvements in perioperative management and patient selection have contributed to enhanced prolonged survival after PD, a minimal invasive approach remains to be found [18–22]. Kamarajah et al. reviewed related articles on different surgical approaches for PD and found that the total laparoscopic approach and the total robotic approach had a lower incidence of wound infections and pulmonary complications and shorter hospitalization time than open approaches [18]. Although Chen et al. demonstrated that minimally invasive PD was technically feasible and safe [23], the outcome of such PD is unclear because it has been argued that the overall morbidity rate might be higher with this procedure than with the traditional open approach [24, 25].

In 2016, ISGPS announced a position statement on pancreatic anastomosis after PD and concluded that no specific approach could eliminate the occurrence of POPF [26]. It was also suggested that no clear differences were found among the open technique, the laparoscopic-assisted approach, the total laparoscopic approach, and the total robotic approach in decreasing the incidence rate of POPF [20]. Instead, consistent practice of any standardized technique and the experience of surgeons may lead to a decrease in this incidence rate [26]. Based on our results, the incidence rate of POPF was slightly lower with modified invaginated anastomosis than with mucosa-to-mucosa anastomosis. The reported incidence rate of POPF was significantly reduced, and the severity of POPF was better discriminated according to the newly announced ISGPS classification [27].

Here, more validation work is still needed due to the limitation of the number of patients. In the future, to promote this improved technique, a multi-center large-scale clinical study would be designed to verify the effectiveness of the modified invaginated pancreaticojejunostomy.

## Conclusion

Modified invaginated pancreaticojejunostomy for PD resulted in a decreased incidence of POPF and thus may be a new approach for PD in the future.

## Data Availability

All data generated or analyzed during this study are included in this published article.
